# Correlation between Chinese visceral adiposity index and serum uric acid levels in type 2 diabetes mellitus patients

**DOI:** 10.3389/fendo.2025.1479662

**Published:** 2025-01-23

**Authors:** Swailla Amina Araújo Intchasso Adotey, Qian Zhang, Mengxue Chen, Yang Jiao, Yin Zhang, Claudette Butoyi, Dong Wang, Ling Yang, Guoyue Yuan, Jue Jia

**Affiliations:** Department of Endocrinology and Metabolism, The Affiliated Hospital of Jiangsu University, Institute of Endocrine and Metabolic Diseases, Jiangsu University, Zhenjiang, China

**Keywords:** type 2 diabetes mellitus, obesity, uric acid, Chinese visceral adiposity index, hyperuricemia

## Abstract

**Purpose:**

The Chinese Visceral Adiposity Index (CVAI), a measure of visceral adiposity dysfunction, is used to assess visceral fat (VFA) malfunction. This research was performed to evaluate the relationship between CVAI and serum uric acid levels in type 2 diabetes mellitus (T2DM) patients.

**Methods:**

A total of 2268 patients with T2DM were enrolled in this study. We collected the general clinical information of patients, measured the basic anthropometric indicators, tested glycolipid metabolism and biochemical indicators, and measured the visceral and subcutaneous fat area with bioelectrical impedance technology. According to the quartiles of the CVAI, the T2DM patients were classified into four groups: group A (CVAI ≤ 94.43), group B (94.43<CVAI ≤ 118.75), group C (118.75<CVAI ≤ 143.95), and group D (CVAI≥143.95), each group has 567 participants. Participants were divided into hyperuricemia (HUA) and non-HUA groups, and the clinical data between the two groups was compared.

**Results:**

Among quartiles of CVAI, as CVAI increased, the proportion of patients with HUA gradually increased. The correlation analysis showed that the majority of basal measures, glycolipid metabolism and biochemical indicators were positively correlated with CVAI. By comparison, the level of CVAI in the HUA group was significantly higher than non-HUA group. Meanwhile, through using the ROC curve, our study observed the more predictive value of CVAI than other obesity indicators for T2DM with HUA.

**Conclusion:**

CVAI is a simple but effective indicator, which is significantly correlated with HUA in T2DM and can reflect the incidence of HUA in T2DM patients. As CVAI increased, the risk of HUA in T2DM patients increased. Therefore, we should pay more attention to the application of CVAI in T2DM.

## Introduction

Diabetes mellitus (DM) is a metabolic condition that has become a major global health concern, due to its high prevalence, associated death, and disability rates (1). DM can be caused by injury to the pancreatic β cell, impaired insulin secretion, or insulin resistance (IR) (2). The International Diabetes Federation (IDF) reported that by 2045, 783.2 million people (12.2% of the global population) would have diabetes, up from 536.6 million adults (10.5%) in 2021 (3). Type 2 diabetes mellitus (T2DM) is a common metabolic condition characterized by a combination of two factors: impaired insulin production by pancreatic β-cells and failure of insulin-sensitive tissues to respond (4).

Obesity is a complex disease influenced by both environmental and genetic factors. The consensus is that overeating and insufficient exercise lead to an imbalance between energy intake and expenditure, which promotes obesity (5). Over the past 40 years, there has been a significant increase in the global prevalence of obesity (6). Obesity has been linked to an increased risk of diabetic complications, particularly abdominal obesity (7). The exact pathophysiological pathways that link obesity to T2DM are still unknown. Several studies have indicated that immune attack induced by overnutrition in multiple organs strongly contributes to IR, lipotoxicity, and glucotoxicity (8). Computed tomography (CT) and Magnetic resonance imaging (MRI) are the most accurate techniques for determining abdominal obesity. However, the cost of the equipment and the ionizing radiation make it impractical to utilize both methods for screening large populations (9). Although many abdominal obesity indices have been established, including waist circumference (WC), visceral adiposity index (VAI), and lipid accumulation product (LAP), their limitation could not be ignored. WC compared to body mass index (BMI), has a better reflection in visceral obesity, while it is hard to distinguish the adipose mass between subcutaneous and visceral fat (10). And because of ethnic difference, VAI is poorly related to adipose tissue in Asian, which is more applied in Caucasians (11). LAP demonstrates greater applicability in white populations, whereas Asian populations tend to accumulate higher amounts of visceral fat, even with relatively low BMI (12).

Chinese Visceral Adiposity Index (CVAI) is a non-invasive measure of visceral adiposity dysfunction that is used to assess the population’s metabolic health (13). It includes high-density lipoprotein cholesterol (HDL-C), BMI, WC, and triglyceride (TG) while accounting for the factors of age and gender. Most remarkably, CVAI has demonstrated its ability to independently predict the development of diabetes and cardiovascular disease, making it a new measure of visceral obesity (14). And visceral fat could be more responsible for hyperuricemia (HUA), which may be resulted from inflammation, insulin resistance, and adipose tissue dysfunction.

Uric acid (UA) is the end product of purine metabolism in humans. If purine metabolism is disrupted, the quantity of UA in the blood might rise, resulting in HUA (15). HUA is the second most common metabolic condition in China, after DM, affecting over 120 million people (16). HUA has historically been linked to gout and nephrolithiasis. It has, meanwhile, also been linked to the onset of cardiometabolic and cardiovascular disorders, as well as T2DM. In T2DM patients, HUA can accelerate the development and deterioration of renal disorders (17). Prior research has demonstrated a link between a higher risk of HUA and being overweight or obese (18). Obesity and HUA likely have a complex relationship where they influence each other through various mechanisms. One way this might happen is HUA can accelerate peripheral lipogenesis and hepatic and cause obesity (19). Nonetheless, obesity is linked to higher levels of UA for several reasons, including decreased renal clearance of UA in obese patients and increased UA synthesis by adipose tissue and xanthine oxidase activity (20). The research that is now available indicates that T2DM, abdominal obesity, and HUA are all related to the etiology of IR. This study aims to investigate the association between CVAI and serum uric acid levels in T2DM patients.

## Methods and material

### Study population

A cross-sectional study was performed on T2DM patients of the Jiangsu University Affiliated Hospital from June 2019 to September 2022. In total 2268 adult patients (1402 male and 866 female) were analyzed in this study. This research complies with the principle of the Helsinki Declaration. The Jiangsu University Affiliated Hospital Ethics Committee approved this study.

The study excluded patients who included the following conditions: type 1 diabetes, gestational diabetes mellitus, special type diabetes, acute or chronic infection (e.g., respiratory infection, intestinal infection and urinary tract infection, etc.), autoimmune disease, hematological disease, chronic lung disease, thyroid dysfunction, those without complete data, history of gout or urate-lowering therapy use, patients with uncontrolled severe medical conditions (e.g., heart failure, severe renal insufficiency).

### Anthropometric and biochemical measurements

The trained survey personnel collected the participants name, gender, age, medication history, and drinking history. Participants removed their hats and shoes, stood upright, and completed the height and weight measurements. With a soft ruler, the trained survey personnel circled the neck through the seventh cervical vertebra to determine neck circumference (NC). To calculate WC, the abdomen was circled along the midpoint of the line connecting the anterior superior iliac spine to the twelfth rib of the mid-axillary line on both sides. The physician used a soft ruler to measure hip circumference (HC) by circling the pubic symphysis and the most convex portion of the buttocks. Using an electronic sphygmomanometer, blood pressure readings were taken after patients were allowed to rest in a quiet place for ten to twenty minutes. Fasting venous blood was taken after an overnight fast of 8h. Fasting plasma glucose (FPG) and 2-h postprandial plasma glucose (2hPG) were determined using the glucose oxidase method. The chemiluminescence method was used to detect fasting plasma insulin (FINS), 2-h postprandial insulin (2hINS). Glycosylated hemoglobin (HbAlc) was determined by high-performance liquid chromatography (HPLC). The liver function was tested by BEKMAN AU5800 automatic biochemical analyzer. The dehydrogenase method was used to detect alanine aminotransferase (ALT); L-γ-glutamyl 3-carboxy-4-nitroaniline method was used to detect γ-glutamyl transpeptidase (γ-glutamyl transpeptidase, γ-GT); The level of total cholesterol (TC), TG, HDL-C, low-density lipoprotein cholesterol (LDL-C), blood urea nitrogen (BUN) and Serum creatinine (Sc) level were measured using enzymatic methods; a fully automatic biochemical instrument was used to measure UA, and a fasting blood uric acid level>420μmol/L can be used to diagnosed with HUA (21).

### Measurement of abdominal visceral fat and subcutaneous visceral fat

The umbilical cord level double bioelectrical impedance analyzer was used to measure the participant’s visceral and subcutaneous fat levels (DUALSCAN; OmronHeathcare Co.Ltd, Kyoto, Japan).

### Indicator calculation

Based on existing data, the following indicators were calculated for subjects:

The BMI calculation formula is as follows (22):


$$\text{BMI}=\text{weight }\left(\text{kg}\right)/{\text{height}}^{2}\;\left({\text{m}}^{2}\right);$$

The homeostatic model assessment of insulin resistance (HOMA-IR) calculation formula is as follows (23):


HOMA-IR=FPG (mmol/L)×FINS (mU/L)/22.5;

The homeostasis model assessment of β-cell function (HOMA-β) calculation formula is as follows:


HOMA-β=20×FINS (mU/L)/[FPG (mmol/L)-3.5];

The WHR calculation formula is as follows (24):


WHR=WC (cm)/HC (cm);

The CVAI calculation using sex-specific formulas is as follows (25):


$$\begin{array}{c} \text{Males}:\text{CVAI}=-267.93\;+\;0.68\times \text{age }\left(\text{years}\right)+0.03\\ \times \text{BMI }\left(\text{kg}/{\text{m}}^{2}\right)+4.00\times \text{WC }\left(\text{cm}\right)+22.00\\ \times \log10\left(\text{TG }\left[\text{mmol}/\text{L}\right]\right)-16.32\\ \times \text{HDL}-\text{C }\left(\text{mmol}/\text{L}\right); \end{array}$$

$$\begin{array}{c} \text{Females}:\text{CVAI}=-187.32\;+\;1.71\times \text{age }\left(\text{years}\right)+4.32\\ \times \text{BMI }\left(\text{kg}/{\text{m}}^{2}\right)+1.12\times \text{WC }\left(\text{cm}\right)+39.76\\ \times \log10\left(\text{TG }\left[\text{mmol}/\text{L}\right]\right)-11.66\\ \times \text{HDL}-\text{C }\left(\text{mmol}/\text{L}\right). \end{array}$$

### Statistical analysis

SPSS Statistics version 29.0 software (SPSS, Inc., Chicago, IL, United States) was used for statistical analyses. A normality test was performed for all parameters before making a statistical analysis. Measurement data by the normal distribution were expressed as Mean ± SD. Non-normally distributed data were presented as median (interquartile range). Student’s *t*-test was used to perform comparisons between the two groups of normal distribution data. The distinction among these four groups was assessed by one-way ANOVA. Mann–Whitney *U* test was taken to compare the two groups of non-normally distributed data. The four groups were analyzed by the Kruskal–Wallis test. Differences in categorical variables were assessed by chi-squared tests. *Pearson* or *Spearman* correlation analysis was performed to examine the relationship between variables. To evaluate the relationship between CVAI and UA, multiple linear regression analyses were conducted. Binary logistic regression analyses were performed to examine to evaluate the relationship between CVAI and HUA. In addition, sensitivity analyses were performed by eliminating unusual outliers. To evaluate the predictive performance of CVAI for the risk of HUA in T2DM, the receiver operating characteristic (ROC) curves were generated. The optimal cut-off values were derived from the Youden index (maximum[sensitivity+specifcity−1]). Finally, chi-squared tests were used to determine differences in the incidence of HUA based on the cutoff values obtained by ROC analysis, and then Cramer’s V was applied to determine the interpreted effect size. All tests of significance were 2-tailed, and *P*<0.05 was considered statistically significant.

## Results

### Baseline characteristics of the study population across CVAI quartiles

According to the quartiles of the CVAI, the included 2268 T2DM patients were classified into four groups (A, B, C and D): group A (CVAI ≤ 94.43), group B (94.43<CVAI ≤ 118.75), group C (118.75<CVAI ≤ 143.95), and group D (CVAI≥143.95), each group has 567 participants. When analyzed by quartiles of CVAI, the patients with higher CVAI were more likely to be male, smokers and drinkers. With respect to medical history, the patients in the higher CVAI were more prone to HTN, hyperlipidemia, CAD and MAFLD. Similar trends were observed for metabolic indices (FINS, 2hINS, HOMA-IR, HOMA- β, TG, ALT, AST, γ-GT, creatinine, and UA) and obesity indices (BMI, NC, WC, HC, WHR, VFA, SFA, and VFA/SFA ratio) (all *P*< 0.001). There were differences in age and LDL-C between the groups (*P*<0.05). However, there were no differences in FPG, 2hPG, HbA1c, TC and BUN between the groups (*P*>0.05) (**Table 1**).

**Table 1:** Baseline characteristics of study population across CVAI quartiles [Mean ± SD, M (Q1, Q3), n (%)].

Variables	Total	A (CVAI ≤ 94.43)	B (94.43<CVAI ≤ 118.75)	C (118.75<CVAI ≤ 143.95)	D (CVAI≥ 143.95)	*P*
N (male/female)	2268 (1402/866)	567 (294/273)	567 (308/259)	567 (360/207)	567 (440/127)	<0.001
Age	56.0 (47.0,63.0)	53.0 (44.0,59.0)	56.0 (49.0,62.0)	57.0 (49.0,65.0)	57.0 (46.0,66.0)	<0.001
Smoking (n, %)	1084 (47.8%)	228 (40.2%)	241 (42.5%)	276 (48.7%)	339 (59.8%)	<0.001
Drinking (n, %)	1083 (47.8%)	233 (41.1%)	256 (45.2%)	277 (48.9%)	317 (55.9%)	<0.001
HTN (n, %)	1468 (64.8%)	238 (42.0%)	366 (64.7%)	407 (71.8%)	457 (80.7%)	<0.001
Hyperlipidemia (n, %)	1847 (81.5%)	380 (67.0%)	446 (78.7%)	502 (88.5%)	519 (91.9%)	<0.001
CAD (n, %)	162 (7.1%)	19 (3.4%)	39 (6.9%)	46 (8.1%)	58 (10.2%)	<0.001
MAFLD (n, %)	1426 (66.00%)	202 (38.2%)	337 (63.0%)	415 (75.7%)	472 (86.1%)	<0.001
HUA (n, %)	315 (13.9%)	35 (6.2%)	49 (8.6%)	94 (16.6%)	137 (24.2%)	<0.001
SBP (mmHg)	128.08 ± 17.60	120.83 ± 15.83	127.79 ± 17.10	129.60 ± 17.65	133.37 ± 17.52	<0.001
DBP (mmHg)	75.51 ± 10.86	72.37 ± 9.98	75.65 ± 11.67	76.09 ± 10.29	78.25 ± 11.01	<0.001
BMI (kg/m^2^)	24.98 ± 3.67	21.75 ± 2.50	24.03 ± 2.22	25.62 ± 2.14	28.66 ± 3.54	<0.001
NC (cm)	38.00 (35.50,40.00)	34.00 (33.00, 36.00)	37.00 (35.00,38.50)	39.00 (37.00,40.00)	41.00 (39.00, 43.00)	<0.001
WC (cm)	90.00 (84.00,97.00)	80.80 (77.00,84.00)	88.00 (85.00,90.00)	93.00 (91.00,95.00)	101.00 (98.00,105.50)	<0.001
HC (cm)	96.05 (92.00,101.00)	91.00 (88.00,95.00)	95.00 (92.00,97.50)	98.00 (95.00,101.00)	103.00 (100.00,107.05)	<0.001
WHR	0.94 ± 0.06	0.88 ± 0.05	0.92 ± 0.05	0.95 ± 0.04	0.98 ± 0.05	<0.001
VFA (cm^2^)	90.69 ± 40.27	54.66 ± 25.87	80.94 ± 25.00	97.85 ± 27.31	128.43 ± 37.32	<0.001
SFA (cm^2^)	183.55 ± 68.06	129.00 ± 44.41	166.70 ± 45.28	192.55 ± 45.69	247.96 ± 69.90	<0.001
V/S	0.50 ± 0.17	0.43 ± 0.18	0.50 ± 0.17	0.52 ± 0.16	0.53 ± 0.14	<0.001
FPG (mmol/L)	9.92 (7.70, 12.72)	10.04 (7.23,12.99)	9.83 (7.47,12.83)	9.98 (8.03,12.47)	9.98 (8.14,12.79)	0.345
2hPG (mmol/L)	18.92 ± 5.29	19.02 ± 5.89	19.10 ± 5.18	19.11 ± 5.05	19.18 ± 4.78	0.840
FINS (µIU/mL)	6.99 (4.15,11.07)	4.82 (2.85,8.39)	6.35 (4.24,9.82)	7.43 (4.46,11.33)	9.79 (6.19,13.75)	<0.001
2hINS (µIU/mL)	26.29 (14.95,43.06)	19.60 (10.52,36.22)	24.90 (14.61,40.40)	28.55 (16.88, 48.02)	33.71 (19.49,49.75)	<0.001
HOMA-IR	2.97 (1.83,4.84)	2.10 (1.23,3.47)	2.71 (1.70,4.35)	3.20 (2.06,5.09)	4.18 (2.71,6.41)	<0.001
HOMA-β	23.17 (11.49,43.94)	17.37 (7.00,36.41)	20.73 (10.89,40.68)	24.16 (12.01,44.86)	28.98 (16.71,51.92)	<0.001
HbA1c (%)	9.70 ± 2.23	9.89 ± 2.55	9.72 ± 2.14	9.50 ± 2.06	9.70 ± 2.00	0.066
TG (mmol/L)	1.88 (1.29, 2.73)	1.30 (0.95, 1.89)	1.87 (1.34,2.65)	2.08 (1.55, 3.06)	2.31 (1.68,3.61)	<0.001
TC (mmol/L)	4.81 (4.17, 5.60)	4.79 (4.05, 5.50)	4.89 (4.23,5.67)	4.82 (4.18, 5.68)	4.79 (4.17,5.56)	0.365
HDL-C (mmol/L)	1.17 (0.97,1.48)	1.37 (1.15,1.70)	1.19 (1.04,1.53)	1.15 (0.93,1.41)	1.03 (0.85,1.22)	<0.001
LDL-C (mmol/L)	2.84 ± 0.93	2.86 ± 0.96	2.93 ± 0.88	2.85 ± 0.88	2.74 ± 0.92	0.005
ALT (U/L)	22.20 (14.30,42.00)	17.80 (11.60,28.40)	21.00 (14.30,40.03)	25.00 (15.00,44.08)	28.70 (18.00,53.90)	<0.001
AST (U/L)	17.70 (13.20,25.00)	15.60 (12.30,21.10)	17.00 (13.10,23.73)	18.00 (13.53,25.25)	19.20 (14.00,29.80)	<0.001
γ-GT (U/L)	29.00 (19.13,50.00)	21.00 (15.00,36.00)	29.00 (19.75,50.00)	30.00 (22.00, 55.75)	35.90 (24.00,60.00)	<0.001
BUN (mmol/L)	5.25 (4.42,6.34)	5.20 (4.39,6.26)	5.28 (4.44,6.29)	5.22 (4.47, 6.28)	5.29 (4.33,6.48)	0.911
Cr (µmol/L)	58.50 (48.90,69.38)	53.50 (45.30,64.00)	59.80 (49.95,68.43)	59.80 (49.95,70.00)	62.20 (53.50,72.90)	<0.001
UA (µmol/L)	294.66 ± 93.66	255.08 ± 81.65	282.97 ± 80.27	306.56 ± 90.78	331.47 ± 93.74	<0.001

HTN, hypertension; CAD, coronary artery disease; MAFLD, metabolic associated fatty liver disease; SBP, systolic blood pressure; DBP, diastolic blood pressure; BMI, body mass index; NC, neck circumference; WC, waist circumference; HC, hip circumference; WHR, waist hip ratio; VFA, visceral fat area; SFA, subcutaneous fat area; V/S, VFA/SFA ratio; FPG, fasting plasma glucose; 2hPG, 2 hour postprandial Plasma Glucose; FINS, fasting insulin; 2hINS, 2 hour postprandial insulin; HOMA- IR, homeostasis model assessment of insulin resistance; HOMA-β, homeostasis model assessment of β-cell function; HbA1c, glycated hemoglobin A1c; TC, total cholesterol; TG, triglyceride; HDL-C, high-density lipoprotein cholesterol; LDL-C, low-density lipoprotein cholesterol; ALT, alanine aminotransferase; AST, aspartate aminotransferase; γ-GT, γ-glutamyl transpeptidase; BUN, blood urea nitrogen; Cr, creatinine; UA, uric acid.

### The prevalence of HUA in T2DM patients across CVAI quartiles

**Figure 1:**
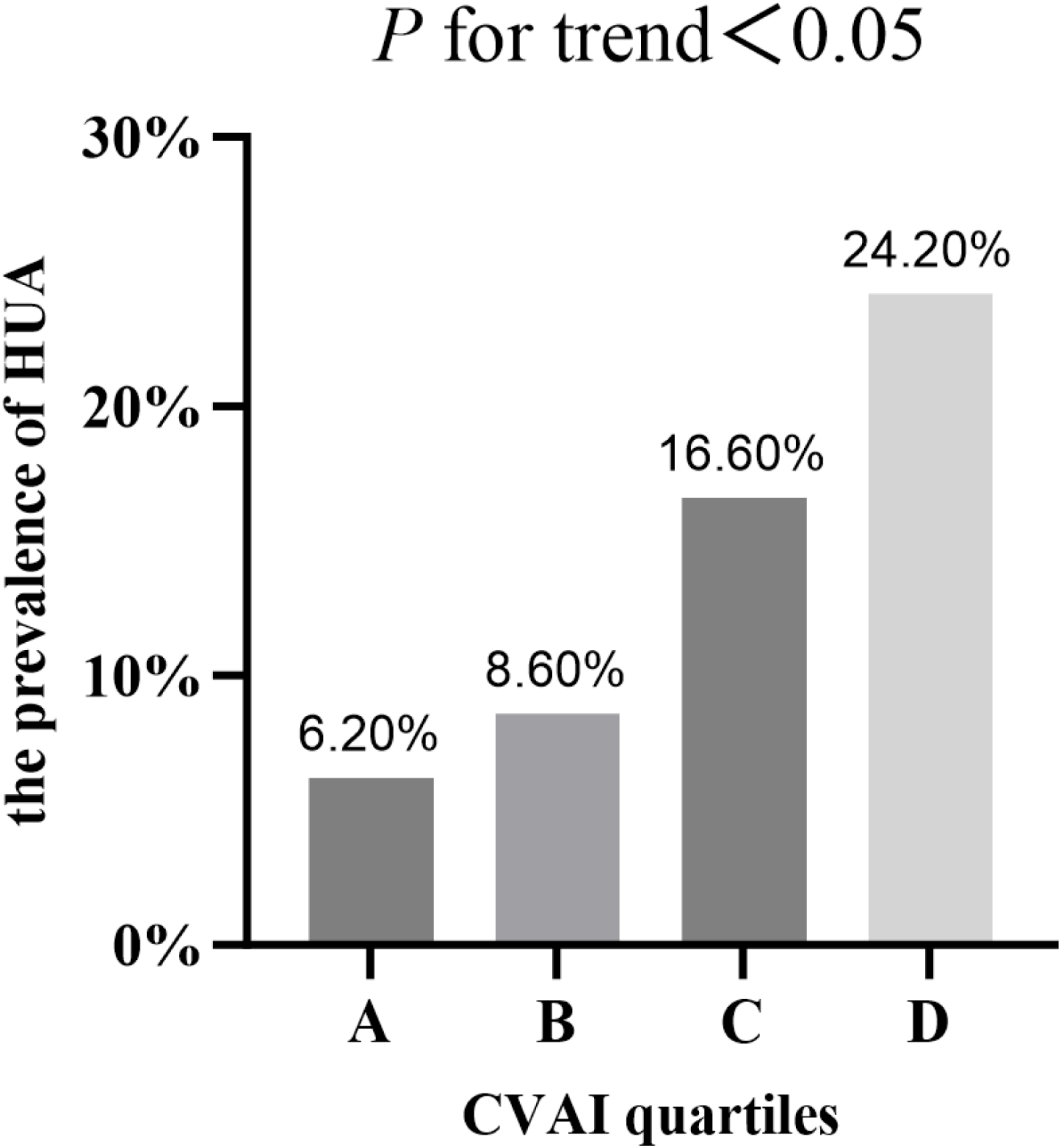
The prevalence of HUA in T2DM patients across CVAI quartiles.

According to the quartiles of the CVAI, the study population was classified into four groups. As shown in **Figure 1**, with the increase of CVAI, the prevalence of HUA gradually increased (*P*<0.05), which were 6.20%, 8.60%, 16.60% and 24.20% respectively (**Figure 1**).

### Correlation of CVAI with other parameters

**Figure 2:**
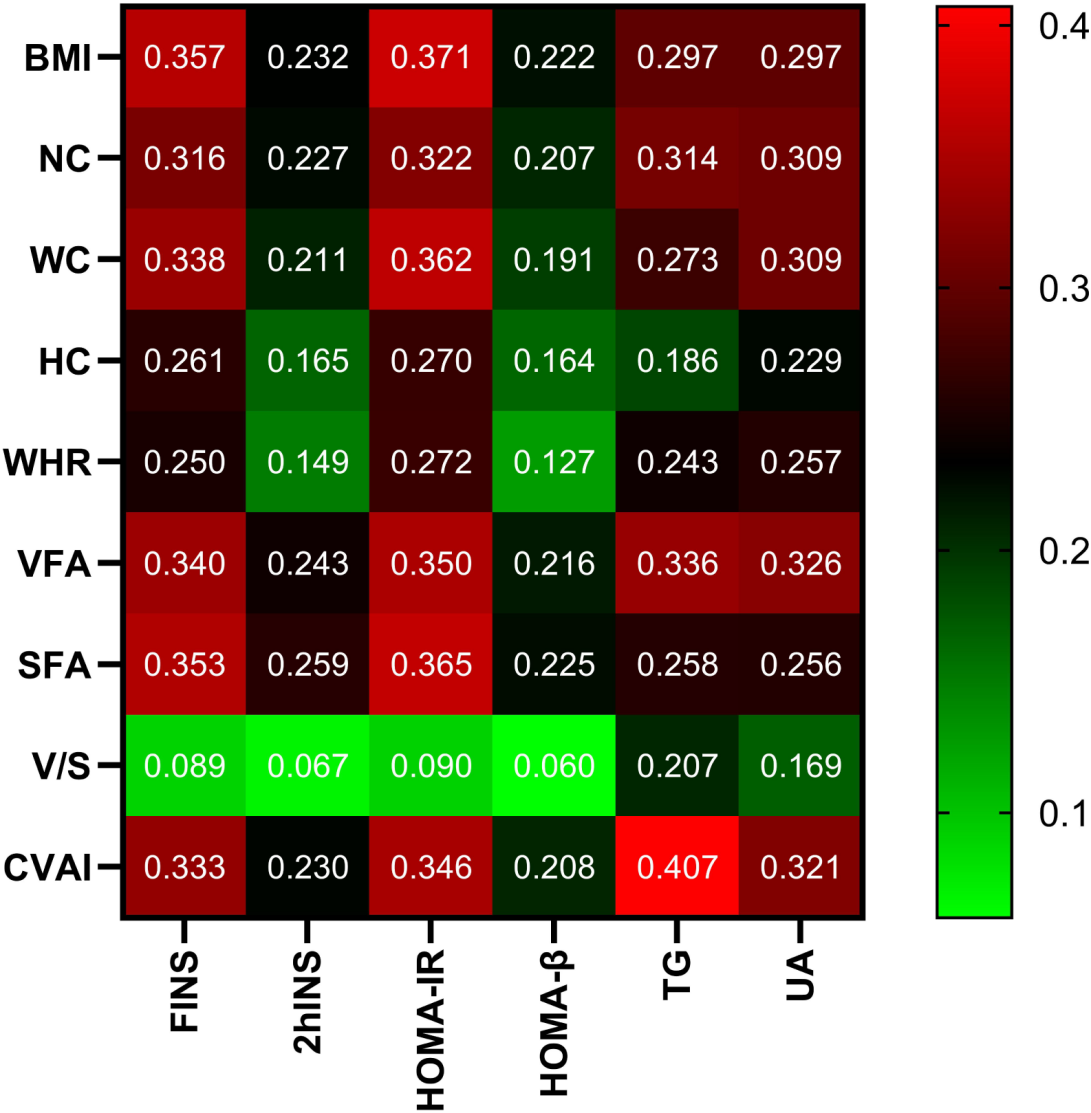
Correlation analysis of body measurements with glucose and lipid metabolism and UA. Note: the color represents the correlation coefficient *r*, and the intensity of the color indicates the level of correlation.

In all participants, CVAI was positively correlated with gender (female=1, male=2), smoking (Yes=1, No=0), drinking (Yes=1, No=0), HTN (Yes=1, No=0), hyperlipidemia (Yes=1, No=0), CAD (Yes=1, No=0), MAFLD (Yes=1, No=0), HUA(Yes=1, No=0), SBP,DBP,NC,HC,VFA,SFA,FINS,2hINS,HOMA-IR,HOMA-β,ALT,AST,γ-GT,Cr and UA (*r*= 0.211,0.158,0.118,0.292,0.259,0.101,0.394,0.206,0.273,0.214,0.691,0.712,0.753,0.725,0.333,0.230,0.346,0.208,0.241,0.178,0.280,0.217,0.319, *P*<0.001, respectively). On the contrary, CVAI was negatively correlated with HbA1c (*r*=-0.055, *P<*0.05). There is no significant correlation between CVAI and FPG, 2hPG, TC, LDL-C, and BUN (*P*>0.05) (**Figure 2**).

### Multiple linear regression analysis with CVAI as dependent variable

With CVAI as the dependent variable, gender, smoking history, drinking history, SBP, DBP, FPG, 2hPG, FINS, 2hINS, TC, LDL-C, ALT, AST, Cr, γ-GT, BUN, NC, HC, WHR, VFA, SFA, V/S, and UA were included in the multiple linear regression equations. It was found that corrected for gender (*β*=16.696, *t*=14.898, 95%CI 14.498~18.894, *P*<0.001,*VIF*=1.270), SBP (*β*= 0.366, *t*=10.034, 95%CI 0.294~0.437, *P*<0.001, *VIF*=1.762), DBP (*β*= -0.469, *t*=-7.893, 95%CI -0.585~-0.352, *P*<0.001, *VIF*=1.814), HC (*β*=1.315, *t*=12.354, 95%CI 1.106~1.524, *P*<0.001, *VIF*=2.607), SFA (*β*=0.171, *t*=12.931 95%CI 0.145~0.197, *P*<0.001, *VIF*=3.466), VFA (*β*=0.351, *t*=19.221, 95%CI 0.315~0.387, *P*<0.001,*VIF*=2.241), AST (*β*=-0.072, *t*=-3.414, 95%CI -0.113~-0.031, *P*<0.001, *VIF*=1.048), CVAI remained independently correlated with UA (*β*=0.020, *t*=3.469, 95%CI 0.009~0.031, *P*<0.001, *VIF*=1.201). For every 1% increase in UA, CVAI increased by 0.020 (**Table 2**).

**Table 2:** Multiple linear regression analysis with CVAI as dependent variable.

Variables	*β*	*T*	*P*	*95%CI*	*VIF*
Gender	16.696	14.898	<0.001	14.498∼18.894	1.270
SBP	0.366	10.034	<0.001	0.294∼0.437	1.762
DBP	-0.469	-7.893	<0.001	-0.585∼-0.352	1.814
HC	1.315	12.354	<0.001	1.106∼1.524	2.607
SFA	0.171	12.931	<0.001	0.145∼0.197	3.466
VFA	0.351	19.221	<0.001	0.315∼0.387	2.241
AST	-0.072	-3.414	<0.001	-0.113∼-0.031	1.048
UA	0.020	3.469	<0.001	0.009∼0.031	1.201

SBP, systolic blood pressure; DBP, diastolic blood pressure; SFA, subcutaneous fat area; VFA, visceral fat area; AST, aspartate aminotransferase; UA, uric acid.

### Characteristics of population by HUA and non-HUA groups

According to the Chinese Multidisciplinary Expert Consensus on the Diagnosis and Treatment of HUA-Related Diseases (2023), the included 2268 T2DM patients were classified into the non-HUA group (n=1,953) and the HUA group (n=315). According to **Table 3**, compared with the non-HUA group, patients in the HUA group had a higher proportion of males, smoking history, drinking history, and CAD, as well as a significantly higher proportion of HTN, hyperlipidemia, and MAFLD (*P<*0.05). Meanwhile, DBP was higher than non-HTN group (*P*<0.001). In addition, there was no significant statistical difference in SPB between the two groups (*P*>0.05). In terms of anthropometric parameters, HUA group had a significant upper level of BMI,NC,WC,HC,WHR,VFA,SFA,V/S and CVAI than non-HUA group (*P*< 0.001).Comparing the biochemical indexes of the two groups, HUA group had a significant upper level of FINS, 2hINS, HOMA-IR, HOMA-β, TG, ALT, AST, γ-GT, BUN, Cr and UA than non-HUA group (*P*<0.001). Meanwhile, there was no significant statistical difference in FPG, 2hPG, HbA1c, and LDL-C between the two groups (*P* > 0.05)(**Table 4**).

**Table 3:** ROC curve analysis of CVAI for risk assessment of HUA in T2DM.

Variables	AUC	95% CI	Sensitivity (%)	Specificity (%)	Cut-of value	Youden index
CVAI	0.674	0.642–0.707	70.3	58.7	123.28	0.290
BMI	0.656	0.624–0.689	62.4	59.3	25.25	0.217
NC	0.664	0.631–0.696	74.5	48.8	37.25	0.233
WC	0.663	0.630–0.695	68.0	57.3	91.45	0.253
WHR	0.620	0.587–0.652	61.0	58.0	0.94	0.181
V/S	0.566	0.534–0.598	83.3	70.6	0.41	0.128

CVAI, Chinese visceral adiposity index; BMI, body mass index; NC, neck circumference; WC, waist circumference; HC, hip circumference; WHR, waist hip ratio; V/S, VFA/SFA ratio.

**Table 4:** Characteristics of population by HUA and non-HUA groups [Mean ± SD, M (Q1, Q3), n (%).

Variables	non-HUA(n=1953)	HUA(n=315)	*P*
Gender(Male/Female)	1953(1157/796)	315(245/70)	<0.001
Age	56(48,63)	52(40,61)	<0.001
Smoking(n, %)	916(46.9%)	168(53.3%)	0.034
Drinking(n, %)	911(46.6%)	172 (54.8%)	0.007
HTN(n, %)	1221(62.6%)	247(78.4%)	<0.001
Hyperlipidemia(n, %)	1555(79.7%)	292(92.7%)	<0.001
CAD(n, %)	130(6.7%)	32(10.2%)	0.025
MAFLD(n, %)	1178(63.5%)	248(81.3%)	<0.001
SBP (mmHg)	127.56 ± 17.52	129.51 ± 18.90	0.103
DBP (mmHg)	75.23 ± 10.79	78.00 ± 12.00	<0.001
BMI (kg/m^2^)	24.70 ± 3.42	27.18 ± 4.42	<0.001
NC (cm)	38.00(35.00,40.00)	40.00(37.50,42.00)	<0.001
WC (cm)	90.00(84.00,96.00)	95.00(90.00,102.00)	<0.001
HC (cm)	96.00(92.00,101.00)	100.00(95.00,106.00)	<0.001
WHR	0.93 ± 0.06	0.96 ± 0.06	<0.001
VFA (cm^2^)	87.57 ± 38.42	111.82 ± 41.33	<0.001
SFA (cm^2^)	172.45(136.50,213.08)	198.80(165.00,258.00)	<0.001
V/S	0.49 ± 0.17	0.53 ± 0.15	<0.001
CVAI	116.43 ± 38.02	143.21 ± 40.95	<0.001
FPG (mmol/L)	10.03(7.68,12.86)	9.88(7.97,12.46)	0.660
2hPG (mmol/L)	19.21 ± 5.16	18.91 ± 5.28	0.263
FINS (µIU/mL)	6.81(3.99,10.82)	9.16(5.79,13.29)	<0.001
2hINS (µIU/mL)	25.65(14.40,42.31)	32.56(19.51,51.53)	<0.001
HOMA-IR	2.87(1.78,4.71)	4.10(2.52,6.26)	<0.001
HOMA-β	22.11(10.85,43.26)	28.67(16.86,51.37)	<0.001
HbA1c (%)	9.77 ± 2.18	9.54 ± 2.18	0.592
TC (mmol/L)	4.91 ± 1.15	5.11 ± 1.36	0.033
TG (mmolL)	1.81(1.25,2.61)	2.64(1.84,4.17)	<0.001
LDL-C (mmol/L)	2.86 ± 0.90	2.76 ± 1.00	0.054
ALT (U/L)	22.00(14.10,40.95)	29.20(17.40,54.30)	<0.001
AST (U/L)	17.25(13.00,24.50)	19.00(14.70,30.75)	<0.001
γ-GT (U/L)	27.25(19.00,47.00)	41.00(26.00,71.50)	<0.001
BUN (mmol/L)	5.22(4.42,6.25)	5.68(4.53,7.09)	<0.001
Cr (µmol/L)	57.10(48.50,67.38)	69.20(57.10,87.00)	<0.001
UA (µmol/L)	270.00(221.00,323.88)	448.50(383.00,488.00)	<0.001

HTN, hypertension; CAD, coronary artery disease; MAFLD, metabolic associated fatty liver disease; SBP, systolic blood pressure; DBP, diastolic blood pressure; BMI, body mass index; NC, neck circumference; WC, waist circumference; HC, hip circumference; WHR, waist hip ratio; VFA, visceral fat area; SFA, subcutaneous fat area; V/S, VFA/SFA ratio; CVAI, Chinese Visceral Adiposity Index; FPG, fasting plasma glucose; 2hPG, 2 hour postprandial plasma glucose; FINS, fasting insulin; 2hINS, 2 hour postprandial insulin; HOMA- IR, homeostasis model assessment of insulin resistance; HOMA-β, homeostasis model assessment of β-cell function; HbA1c, glycated hemoglobin A1c; TC, total cholesterol; TG, triglyceride; HDL-C, high-density lipoprotein cholesterol; LDL-C, low-density lipoprotein cholesterol; ALT, alanine aminotransferase; AST, aspartate aminotransferase; γ-GT, γ-glutamyl transpeptidase; BUN, blood urea nitrogen; Cr, creatinine.

### Comparison of CVAI between HUA and non-HUA groups

**Figure 3:**
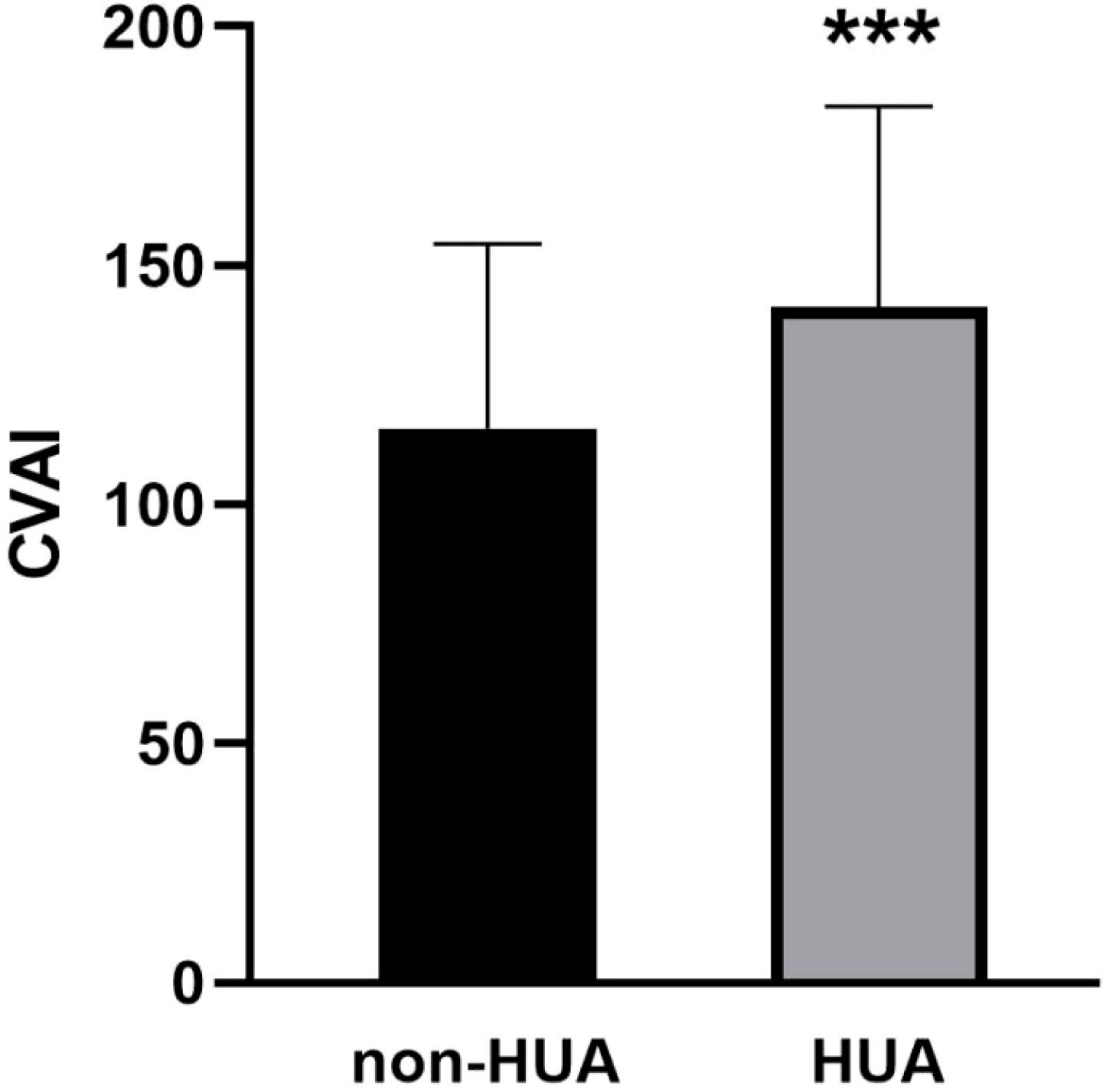
Comparison of CVAI between HUA and non-HUA groups. *** represent *P*<0.001.

As shown in **Figure 3**, the baseline level of CVAI in the HUA group was significantly higher (143.21 ± 40.95), compared to the non-HUA group (116.43 ± 38.02) (*P* < 0.001).

### Comparison of CVAI between genders and ages in different groups

**Figure 4:**
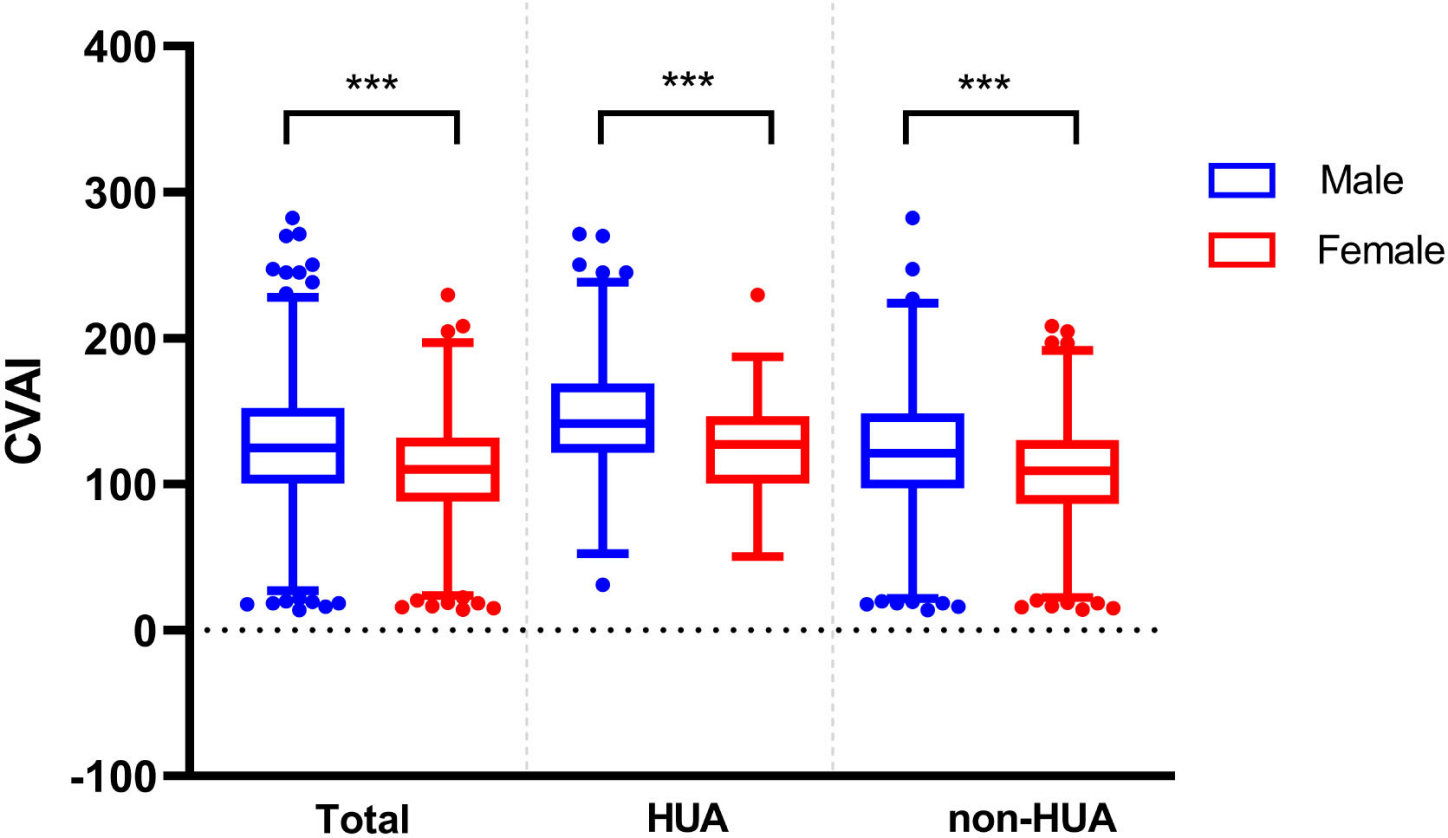
Comparison of CVAI between different genders in the two groups. *** represent *P*<0.001.

In the patients with T2DM, the baseline CVAI level was significantly higher in the male group (125.24 ± 41.33) than in the female group (108.53 ± 35.28) (*P* < 0.001). And we found the same conclusion in patients with T2DM combined with HUA (male: 146.58 ± 42.78, female: 122.71 ± 33.13) and in patients with non-HUA (male: 121.93 ± 39.73, female: 107.28 ± 35.21) (*P* < 0.001) (**Figure 4**).

**Figure 5:**
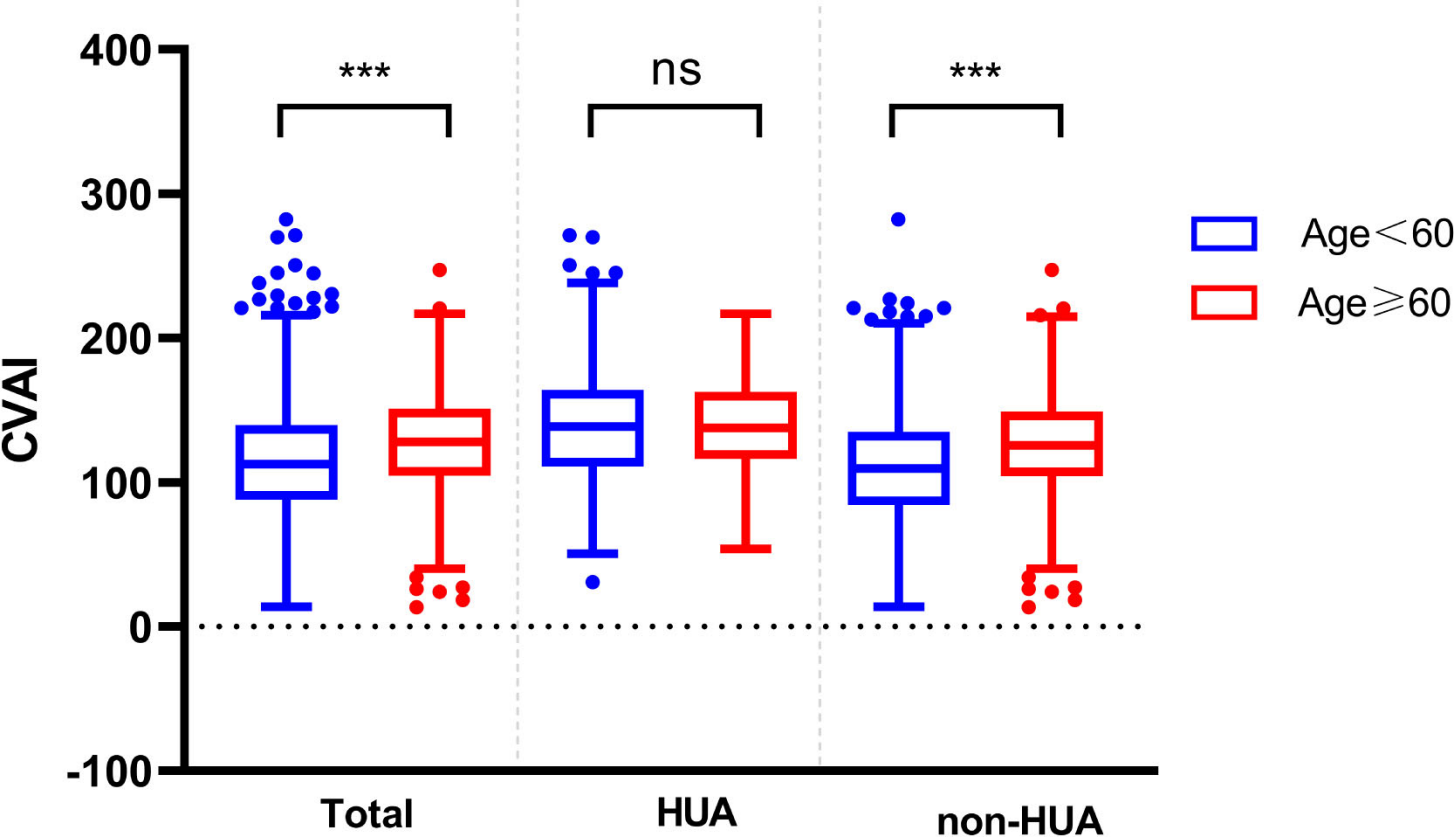
Comparison of CVAI between different ages in the two groups. ns represent *P>*0.05, *** represent *P<*0.001.

At the same time, the differences in CVAI were observed among different age groups. In the total population and non-HUA group, the CVAI value of patients less than 60 years old was lower than that of above 60 years old patients (*P* < 0.001) (**Figure 5**).

The sensitivity analyses showed similar difference to the primary analyses (**Supplementary Figures 1, 2**).

### ROC curves of CVAI in predicting HUA risk among all population and stratified by age

**Figure 6:**
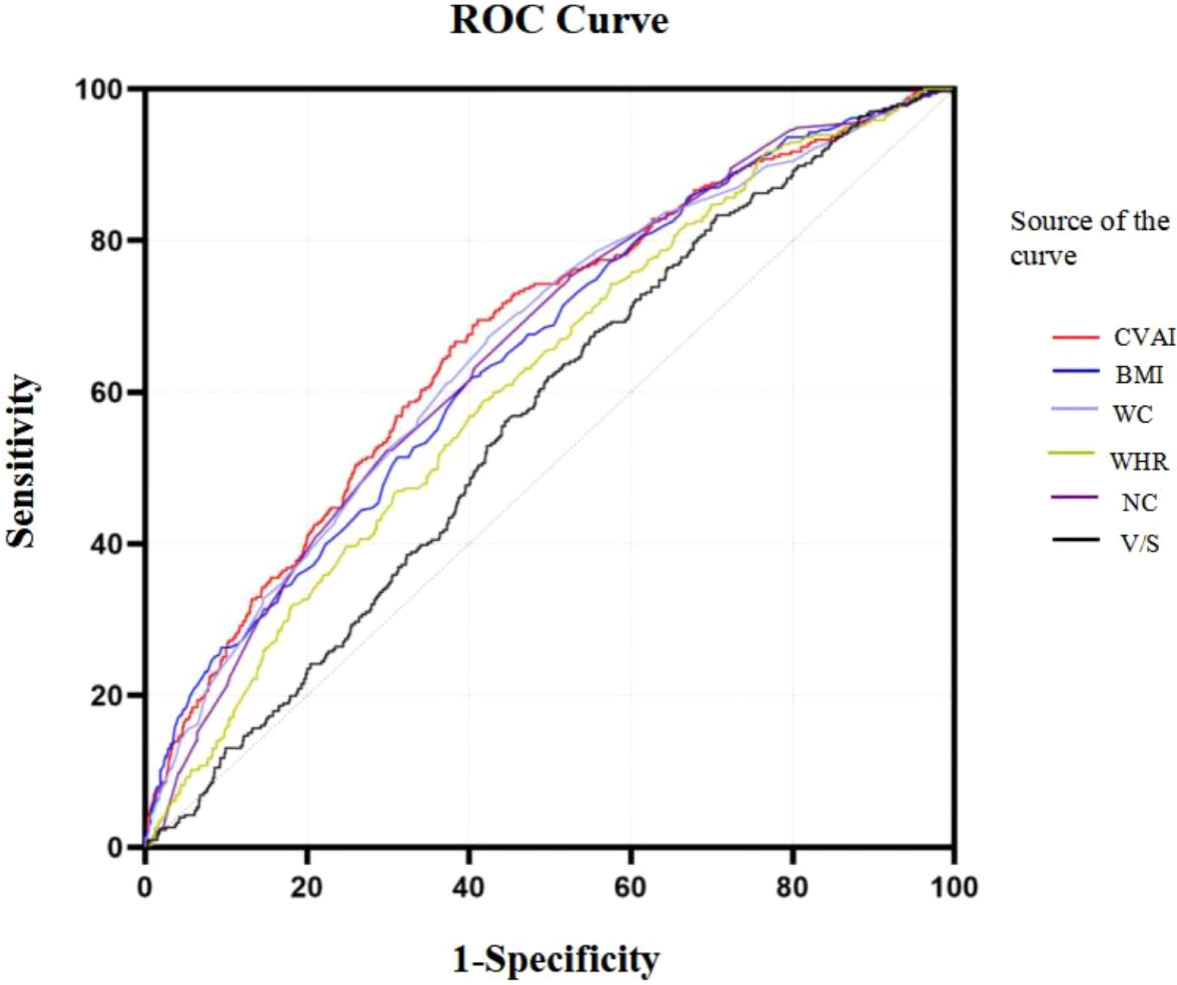
ROC curves of CVAI in predicting HUA risk among all population.

Based on the results of the ROC analysis as presented in **Figure 6**, the analysis showed the diagnostic ability of obesity indicators including BMI, NC, WC, WHR, V/S and CVAI in T2DM with HUA. The area under the ROC curve of BMI, NC, WC, WHR, V/S and CVAI was 0.656, 0.664, 0.663, 0.620, 0.566 and 0.674, respectively. Compared with other body measurements, CVAI had the largest area under the ROC curve, which was 0.674 (95%CI: 0.642 –0.707), and the cut-off value of CVAI was 123.28, the sensitivity was 70.3%, and the specificity was 58.7% (**Table 3**) (**Figure 6**). Also, we used binary logistic regression analyses to examine this conclusion (**Supplementary Table 1**).

Results of the ROC curves analysis of the adiposity indicators to predict HUA risk in ≥60 years old and <60 years old participants are shown in **Supplementary Figure 3**. In ≥60 years old participants, CVAI had the highest AUC (0.615), followed by NC (0.601), HOMA-IR (0.587), BMI (0.542). In <60 years old participants, CVAI had the highest AUC (0.708), followed by BMI (0.698), NC (0.681), HOMA-IR (0.630) (all *P*< 0.05) (**Supplementary Tables 2, 3**) (**Supplementary Figure 3**).

### Incidence of HUA according to CVAI cutoff value

**Figure 7:**
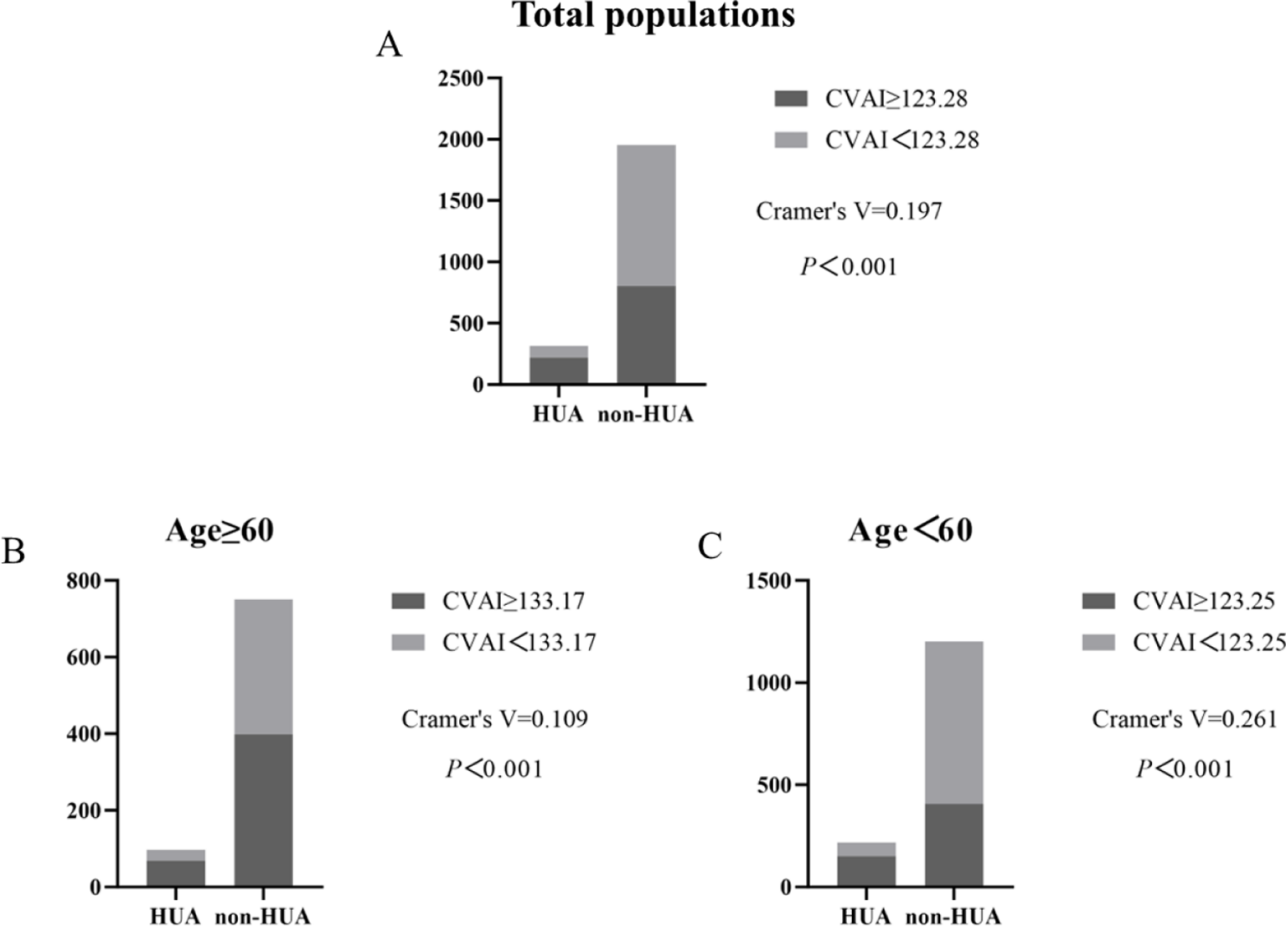
Incidence of HUA according to CVAI cutoff value in total populations, ≥60 years old and <60 years old groups. **(A–C)**.

Compared by CVAI cutoff value, participants with high levels of CVAI had a significantly higher risk of HUA in both ≥60 years old and <60 years old groups (all *P*< 0.001) (**Figure 7**).

## Discussion

Obesity is an excess of body fat brought on by a chronically insufficient energy balance, a multifactorial and complex condition (26). Researchers showed that reduced adipose tissue oxygenation during obesity can increase the concentration of plasma plasminogen activator inhibitor-1 concentrations, increase plasma levels of branched-chain amino acids, induce IR, and promote the incidence of T2DM (27). Furthermore, obesity induces the accumulation of immune cells and increased polarization of inflammation, thereby increasing metabolic dysfunction in numerous tissues such as the liver, adipose tissue, gut, and skeletal muscle, eventually contributing to the formation of IR and T2DM (28). Obesity and HUA are closely related, with multiple studies indicating a link between BMI and the risk of HUA (29–31). According to reports, the main contributing cause of HUA is visceral fat (32). Compared with BMI, CVAI is more associated with visceral obesity and HOMA-IR (33). In obesity, adipose tissue tends to be hypoxic, leading to adipose tissue dysfunction, which is manifested as dysregulation of adipocytokines and chronic low-grade inflammation. Meanwhile, hypoxic adipose tissue could increase the activity of xanthine oxidoreductase (XOR) and result in more secretion of UA (20). Furthermore, due to the enhanced lipolysis of visceral fat, visceral fat tissue could generate more flow of free fatty acids (FFA) towards the liver, which in turn accelerates the pentose phosphate pathway and induces the increase metabolism of purine (34). Sun et al. discovered that independent of BMI classification, adipose tissue insulin resistance index (Adipo-IR) is highly correlated with serum UA and HUA. Adipo-IR is more closely linked to HUA in men with normal BMI than HOMA-IR (23). IR in adipose tissue can lead to compensatory hyperinsulinemia, which reduces UA clearance in the kidneys and ultimately result in HUA (35).

UA is the final result of the human body’s metabolism of purine nucleotides. UA production and excretion in the body are balanced under normal conditions. HUA develops when this equilibrium is upset (36). Studies have shown a correlation between elevated serum uric acid levels and prevalent medical disorders such as obesity, IR, diabetes, and metabolic syndrome (37). Recently, UA has been considered a risk factor for T2DM. Because IR is exacerbated and insulin secretion is stimulated by HUA (38).

This study included a total of 2268 T2DM patients and explored the association between CVAI and serum uric acid levels in T2DM patients. According to the CVAI quartile grouping, the proportion of males, smoking, drinking, HTN, hyperlipidemia, CAD, MAFLD, HUA, SBP, DBP, etc. gradually increased. Wang et al. also found that there is a strong correlation between elevated CVAI and a higher risk of carotid plaque and cardiovascular disease (CVD) (39). Similarly, Cai et al. reported a notable independent nonlinear association between CVAI and the incidence of new-onset myocardial infarction (MI) (40). This indicates that visceral adiposity, as measured by CVAI, may be a significant contributor to predicting cardiometabolic complications in T2DM patients.

The study also found that the anthropometric parameters, glucose metabolism indicators, and abdominal obesity indicators of the HUA group were higher than those of the non-HUA group. As for the CVAI value, the HUA group was higher than the non-HUA group. The study showed a significant correlation between CVAI and visceral fat area. We also observed a positive correlation between CVAI and various cardiometabolic risk factors in patients with T2DM. After adjusting for confounding factors, CVAI remains an independent risk factor for HUA in the T2DM population.

Previous studies have shown that VAF and serum UA concentration are highly correlated in individuals recently diagnosed with T2DM. The former is recognized as a stand-alone risk factor for HUA and a useful marker for determining whether HUA is present and predicting IR (41). This study also found a correlation between visceral adiposity and HUA in patients with T2DM, suggesting a potential link between VAF accumulation and UA metabolism in T2DM patients.

The results of statistical analysis in our study showed that the prevalence of HUA gradually increased with the increase of CVAI. We also found that CVAI is higher in males with T2DM compared to females, regardless of whether they have HUA. And meanwhile we made a discovery that men exhibit a higher propensity to develop HUA. This disparity might potentially be attributed to the notably elevated rates of alcohol and tobacco consumption among men. Additionally, Zhang et al. also described that estrogen, an effective uricosuric agent, appeared to neutralize the adverse effects induced by CVAI on the augmented risk of HUA (42). Patients with T2DM had a greater incidence of HUA, which may be caused by various factors commonly found in T2DM, including increased body weight, WC, dyslipidemia, sedentary lifestyle, HTN, and IR. According to studies HUA has been linked to proteinuria and may be involved in the etiology of diabetic microvascular disorders (43). People with T2DM and prediabetes typically have greater serum UA levels than people without these disorders (44).In this study, it was also found that the FINS and 2hINS in the HUA group were higher than the non-HUA group, which further illustrated that there was a connection between IR and HUA.

This study also explored the CVAI as an indicator to predict the risk of HUA in the general population. The data indicate that CVAI has some potential, although it is not without limitations. The area under the ROC curve (AUC) for CVAI was 0.672 (95% CI: 0.640-0.704), indicating moderate discriminatory power for identifying individuals at risk of HUA. We also found that CVAI behaved slightly better in HUA prediction than other adiposity indicators such as BMI, WC, WHCR, NC, HC, and VFA. More research is needed to compare CVAI performance to existing HUA risk variables or scoring methods.

We studied the relationship between CVAI and UA in T2DM and concluded that, compared with other common obesity indicators, CVAI can better predict HUA. In clinical practice, we can advise the patients to lose weight and decrease lipid to prevent the HUA based on CVAI. However, our research has some limitations. First, our study only targets Chinese population, and a multicenter study including participants from non-Asian countries should be conducted in the future. Second, serum UA is affected by other confounding factors, such as dietary habits and physical activity, which we will describe in detail in future research. And although the accuracy of bioelectrical impedance technology, which is used to measure abdominal obesity, is poorer than CT or MRI, it is a simple and economical method, and it has been used widely in clinical practice. In the future, we will use longitudinal studies to further explain the relationship between CVAI and UA levels in T2DM patients and verify the clinical utility of CVAI in predicting HUA.

## Conclusion

In summary, CVAI is a simple indicator that can be used to predict the risk of HUA in T2DM patients in the Chinese population, and uric acid level is worth paying attention to during the diagnosis and treatment of T2DM patients.

## Data Availability

The raw data supporting the conclusions of this article will be made available by the authors, without undue reservation.
