# Supplementary material for: Correlation between Chinese visceral adiposity index and serum uric acid levels in type 2 diabetes mellitus patients
Source: Front Endocrinol (Lausanne). 2025 Jan 23;16:1479662. doi: 10.3389/fendo.2025.1479662 (PMC11798813; doi:10.3389/fendo.2025.1479662)
Supplement: Supplementary file 1 [file DataSheet1.pdf]

Supplement Table 1 Binary logistic regression analyses of CVAI for assessment of HUA in T2DM

Variable	*β*	SE	OR (95%CI)	*P*
CVAI	0.014	0.004	1.014 (1.005~1.022)	0.001
BMI	0.088	0.036	1.092 (1.017~1.172)	0.015
NC	0.061	0.048	1.063 (0.967~1.169)	0.208
WC	-0.040	0.022	0.961 (0.920~1.004)	0.072
WHR	1.333	1.565	3.792 (0.176-81.537)	0.394
V/S	-0.005	0.665	0.995 (0.270-3.667)	0.994

Note: CVAI: Chinese visceral adiposity index; BMI: body mass index; NC: neck circumference; WC: waist circumference; HC: hip circumference; WHR: waist hip ratio; V/S: VFA/SFA ratio

Supplement Table 2 Age<60 ROC curve analysis of CVAI for risk assessment of HUA in T2DM

Variables	AUC	95% CI	Sensitivity (%)	Specificity (%)	Cut-off value	Youden index
CVAI	0.708	0.671–0.746	69.3	66.1	123.25	0.354
BMI	0.698	0.661–0.735	69.3	59.5	25.25	0.288
NC	0.681	0.644–0.719	68.3	59.7	38.25	0.280
HOMA-IR	0.630	0.587–0.673	58.9	63.3	3.64	0.222

Note: CVAI: Chinese visceral adiposity index; BMI: body mass index; NC: neck circumference; HOMA- IR: homeostasis model assessment of insulin resistance

Supplement Table 3 Age≥60 ROC curve analysis of CVAI for risk assessment of HUA in T2DM

Variables	AUC	95% CI	Sensitivity (%)	Specificity (%)	Cut-off value	Youden index
CVAI	0.615	0.554–0.676	62.9	60.2	133.17	0.231
BMI	0.542	0.482–0.602	23.7	84.7	27.65	0.084
NC	0.601	0.543–0.659	67.0	48.3	37.80	0.153
HOMA-IR	0.587	0.528–0.646	77.8	42.7	2.48	0.205

Note: CVAI: Chinese visceral adiposity index; BMI: body mass index; NC: neck circumference; HOMA- IR: homeostasis model assessment of insulin resistance;

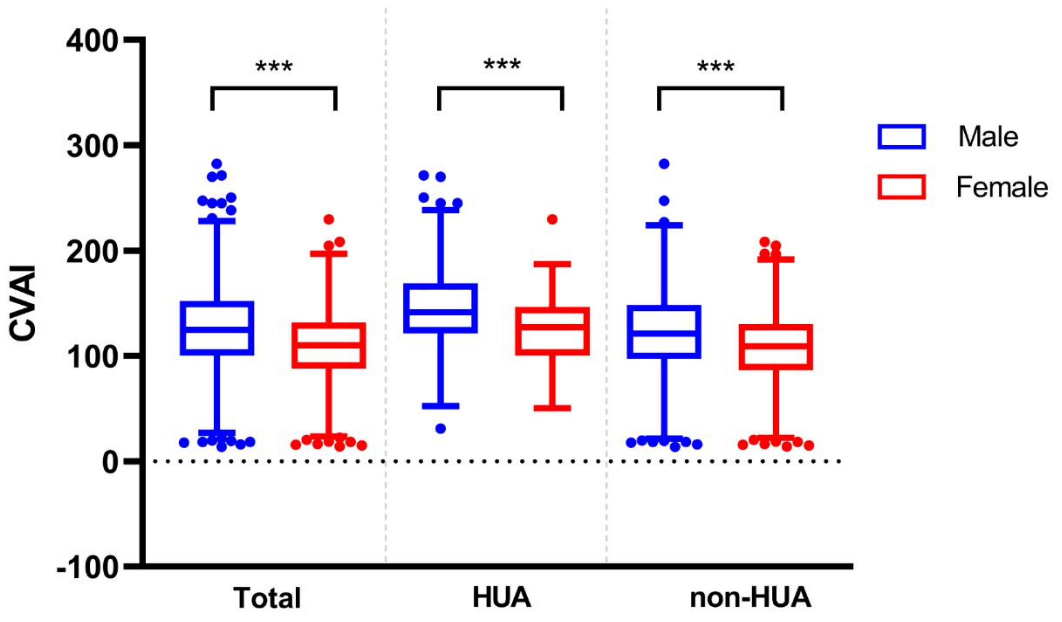

Supplement Fig.1 Comparison of CVAI between different genders in the two groups
Note： *** represent P<0.001

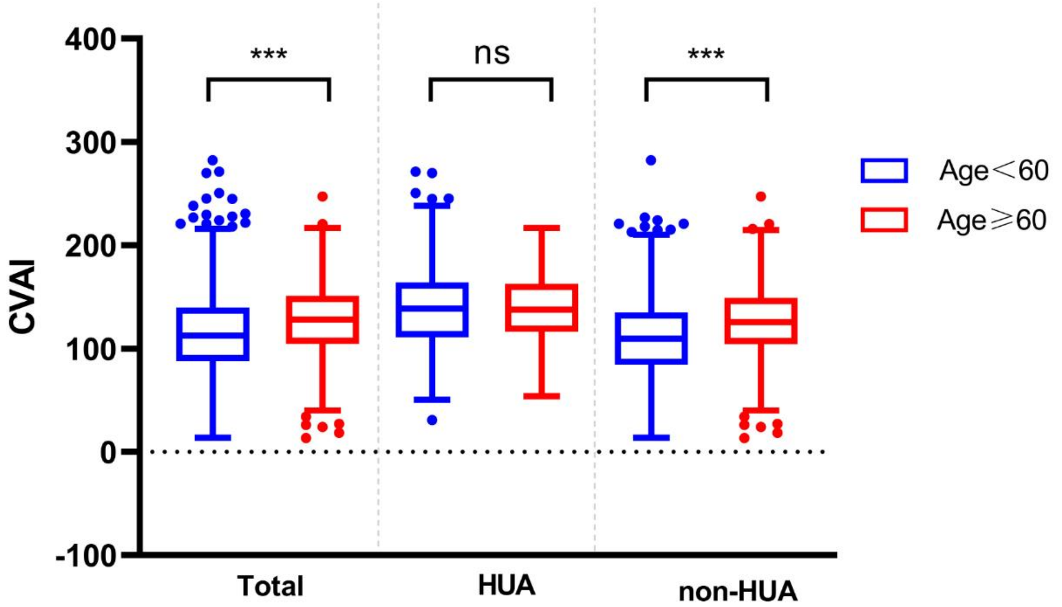

Supplement Fig.2 Comparison of CVAI between different ages in the two groups
Note: Ns represent P >0.05, *** represent P <0.001

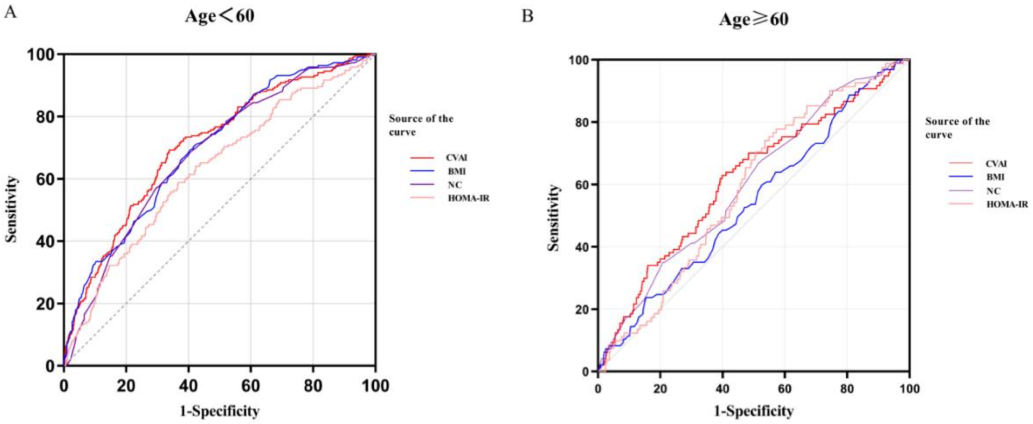

Supplement Fig.3 Comparison of the predictive value of adiposity indices for diagnosis of incident HUA in different age

Note: HUA, hyperuricemia; CVAI: Chinese Visceral Adiposity Index; BMI: body mass index; NC: neck circumference; HOMA- IR: homeostasis model assessment of insulin resistance;
